# Supplementary material for: Pathological mutations reveal the key role of the cytosolic iRhom2 N-terminus for phosphorylation-independent 14-3-3 interaction and ADAM17 binding, stability, and activity
Source: Cell Mol Life Sci. 2024 Feb 27;81(1):102. doi: 10.1007/s00018-024-05132-3 (PMC10896983; doi:10.1007/s00018-024-05132-3)
Supplement: Supplementary file 1 — Supplementary file1 (PDF 1022 KB) [file 18_2024_5132_MOESM1_ESM.pdf]

Figure S1

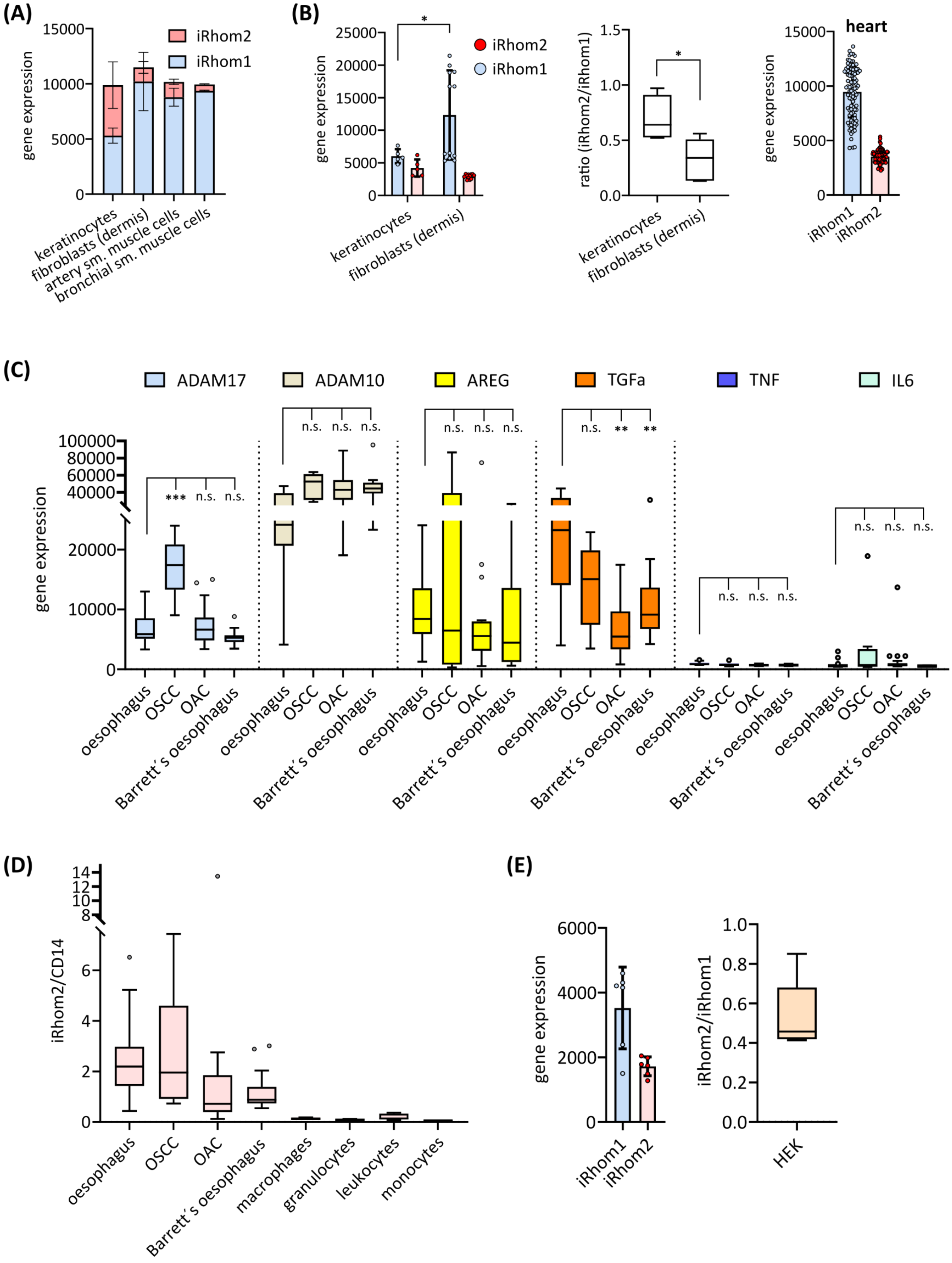

(A)
iRhom2
iRhom1
gene expression
keratinocytes
fibroblasts (dermis)
artery sm. muscle cells
bronchial sm. muscle cells
(B)
iRhom2
iRhom1
gene expression
keratinocytes
fibroblasts (dermis)
ratio (iRhom2/iRhom1)
heart
iRhom1
iRhom2
(C)
ADAM17
ADAM10
AREG
TGFa
TNF
IL6
gene expression
n.s.
***
**
oesophagus
OSCC
OAC
Barrett´s oesophagus
(D)
iRhom2/CD14
oesophagus
OSCC
OAC
Barrett´s oesophagus
macrophages
granulocytes
leukocytes
monocytes
(E)
gene expression
iRhom1
iRhom2
iRhom2/iRhom1
HEK

Figure S1: (A) Gene expression levels of iRhom1 and iRhom2 (see Fig. 1C) from primary cell samples are shown as stacked bars, indicating that total iRhom (iRhom1 plus iRhom2) expression may be regulated to a similar overall level in untreated primary cells from healthy tissue. **(B)** Gene expression levels of iRhom1 and iRhom2 from untreated primary murine keratinocytes from healthy tissue (n = 5), untreated primary murine fibroblasts from healthy dermis tissue (n = 12) and samples from healthy murine heart tissue (n = 98) were analysed. **(C)** Gene expression levels of ADAM17, AREG, TGFα, TNFα and IL6 were analysed in oesophagus and cancerous oesophagus tissues (see Fig. 6C). **(D)** To test whether the increased iRhom2 expression levels were due to the presence of macrophages or monocytes in the samples, the ratio between iRhom2 and CD14 (monocyte and macrophage markers) of the samples was calculated and compared with the values of the indicated immune cell populations (Fig. S2), showing that the measured high iRhom2 levels cannot be explained by the presence of immune cells.**(E)** Gene expression levels of iRhom1 and iRhom2 fromHEK293 cells (n = 6).

Figure S2

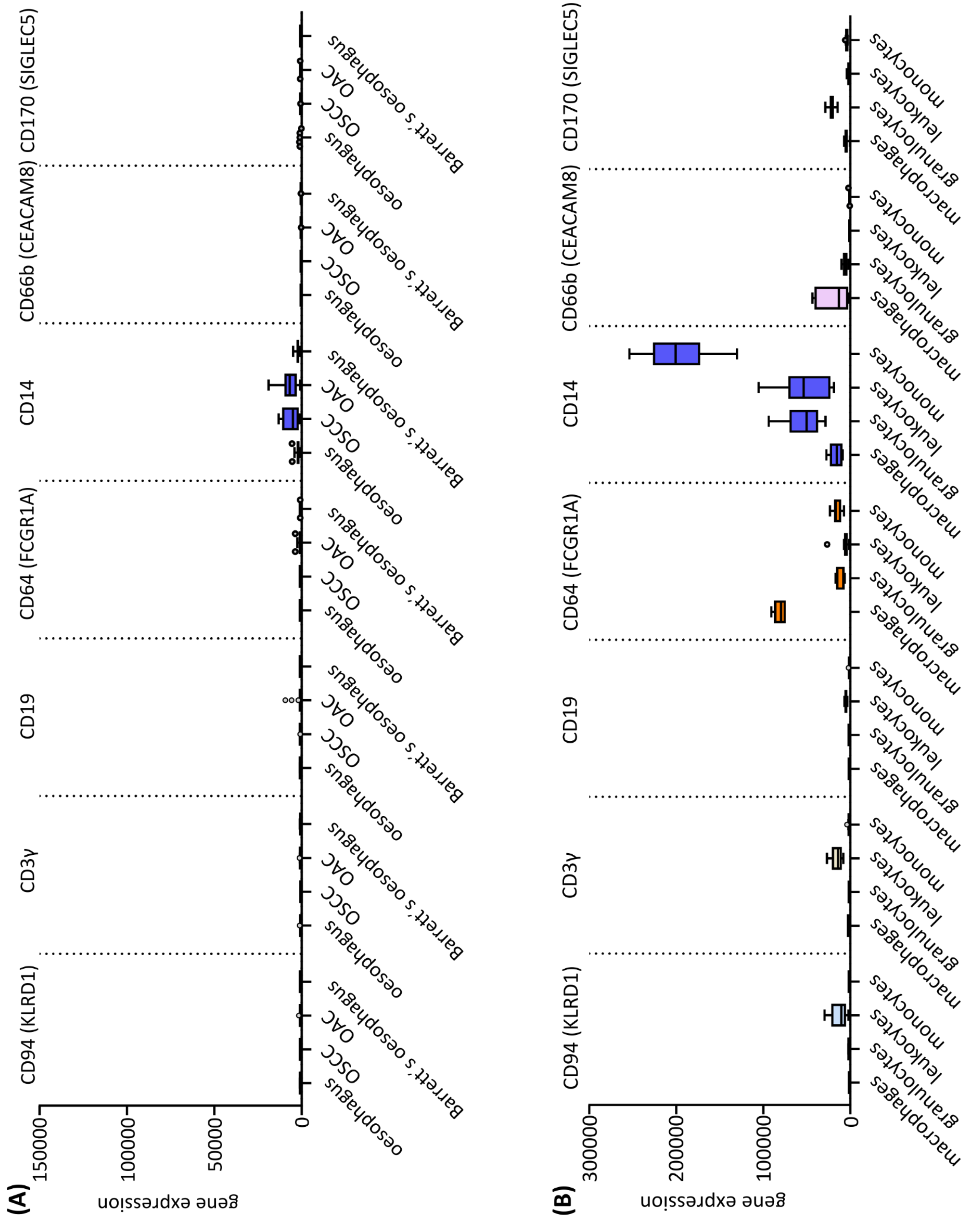

(A)
gene expression
150000
100000
50000
0
CD94 (KLRD1)
CD3γ
CD19
CD64 (FCGR1A)
CD14
CD66b (CEACAM8)
CD170 (SIGLEC5)
oesophagus
OSCC
OAC
Barrett´s oesophagus
(B)
gene expression
300000
200000
100000
0
macrophages
granulocytes
leukocytes
monocytes

Figure S2: Gene expression levels of indicated immune cell markers: CD94 (NK-cells), CD3γ (T-cells), CD19 (B-cells), CD64 (monocytes/macrophages, Dendritic cells), CD14 (monocytes/macrophages, granulocytes), CD66b/CD67 (granulocytes), CD170 (tumour-associated neutrophiles, monocytes/macrophages, activated dendritic cells, lymphocytes-subsets) were analysed with the GENEVESTIGATOR platform **(A)** in oesophagus and cancerous oesophagus tissues (see Fig. 1G) and **(B)** in indicated human immune cell populations: macrophages (n = 6), granulocytes (n = 6), leukocytes (n = 8) and monocytes (n = 13).

Figure S3

(A)

```
muiR2          1 MASADKNGSNLPSVSGSRLQSRKPPNLSITIPPP--ESQAPGEQDSMLPE----------
huiR2iso1      1 MASADKNGGSVSSVSSSRLQSRKPPNLSITIPPPEKETQAPGEQDSMLPEGFQNRRLKKS
huiR2iso2      1 MASADKNGGSVSSVSSSRLQSRKPPNLSITIPPPEKETQAPGEQDSMLPE----------
                 ********..:.***.******************  *:************

muiR2         61 ------------------RRKNPAYLKSVSLQEPRGRWQEGAEKRPGFRRQASLSQSIRK
huiR2iso1     61 QPRTWAAHTTACPPSFLPKRKNPAYLKSVSLQEPRSRWQESSEKRPGFRRQASLSQSIRK
huiR2iso2     61 -------------------RKNPAYLKSVSLQEPRSRWQESSEKRPGFRRQASLSQSIRK
                                    ****************.****.:******************
```

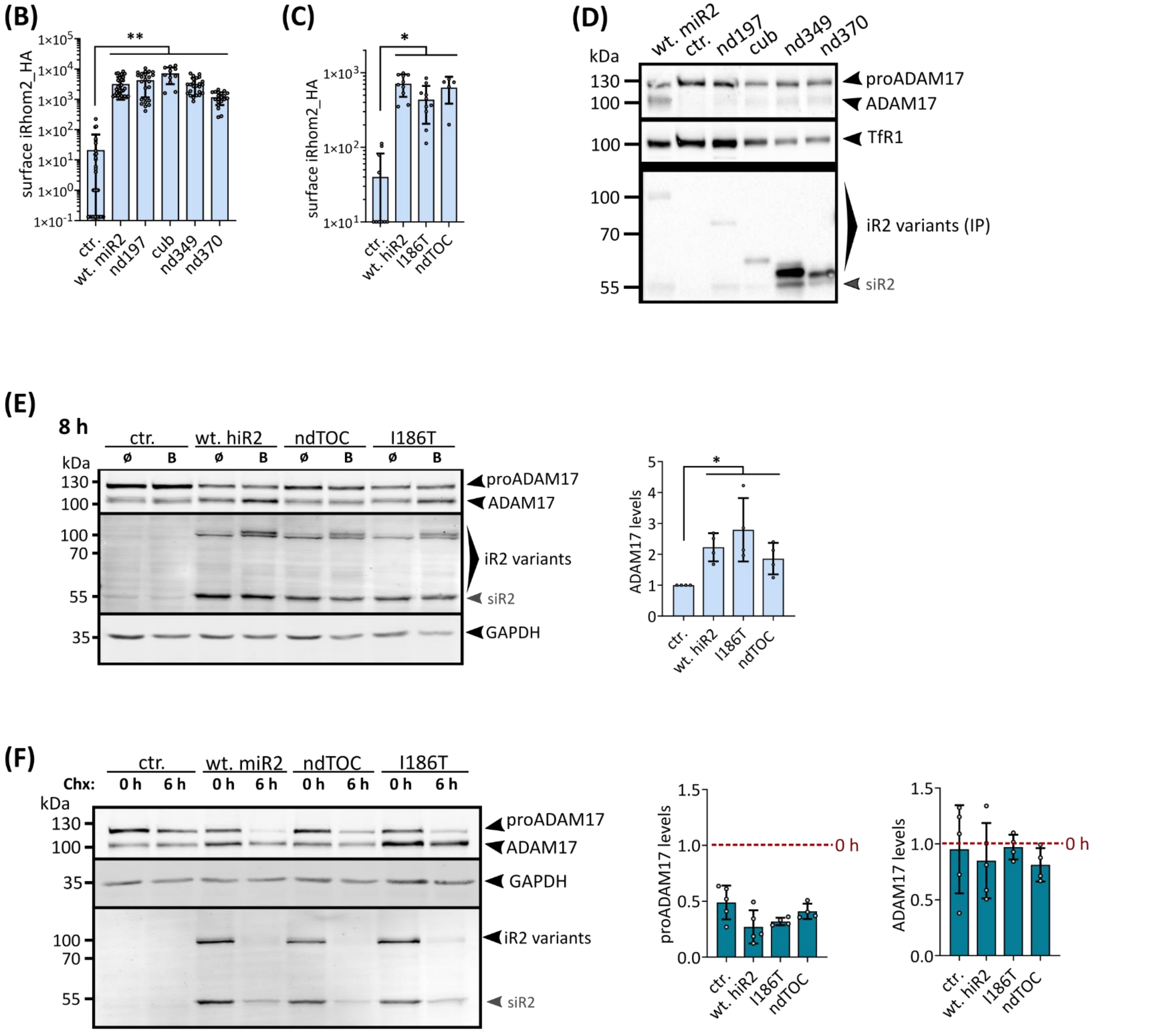

Figure S3: (A) Protein sequence alignment of the N-terminal part of the cytosolic IDR of human iRhom2 (isoform 1 and isoform 2) and murine iRhom2. **(B)** Surface localisation of iRhom2 variants (with HA tag) in cells stably expressing the indicated murine constructs were measured by flow cytometry. For quantification, the geometric mean of the specific fluorescence signal was determined. n > 11. **(C)** Surface localisation of iRhom2 variants (with HA tag) in cells stably expressing the indicated human constructs were measured by flow cytometry. For quantification, the geometric mean of the specific fluorescence signal was determined. n > 5. **(D)** Immunoblot of samples from MEFs deficient for iRhom1 and iRhom2 stably expressing the indicated iRhom2 variants or GFP (ctr.) were used. The transferrin receptor (TfR1) served as an input control. (n = 3). **(E)** To analyse the fate of ADAM17 in cells expressing the indicated TOC mutations, cells were treated with bafilomycin ("B") for 8 h to inhibit lysosomal degradation. Levels of ADAM17 were assessed by densitometric measurements and calculation of the ratio between ADAM17 and the loading control GAPDH, which is independent of lysosomal degradation. (n > 4). **(F)** To assess the turn-over rate of ADAM17 a cycloheximide-based (Chx) pulse-chase experiment was performed. Reduction of ADAM17 levels was assessed by densitometric measurements and normalisation to 0 h Chx treatment (set to 1). (n = 5).

Figure S4

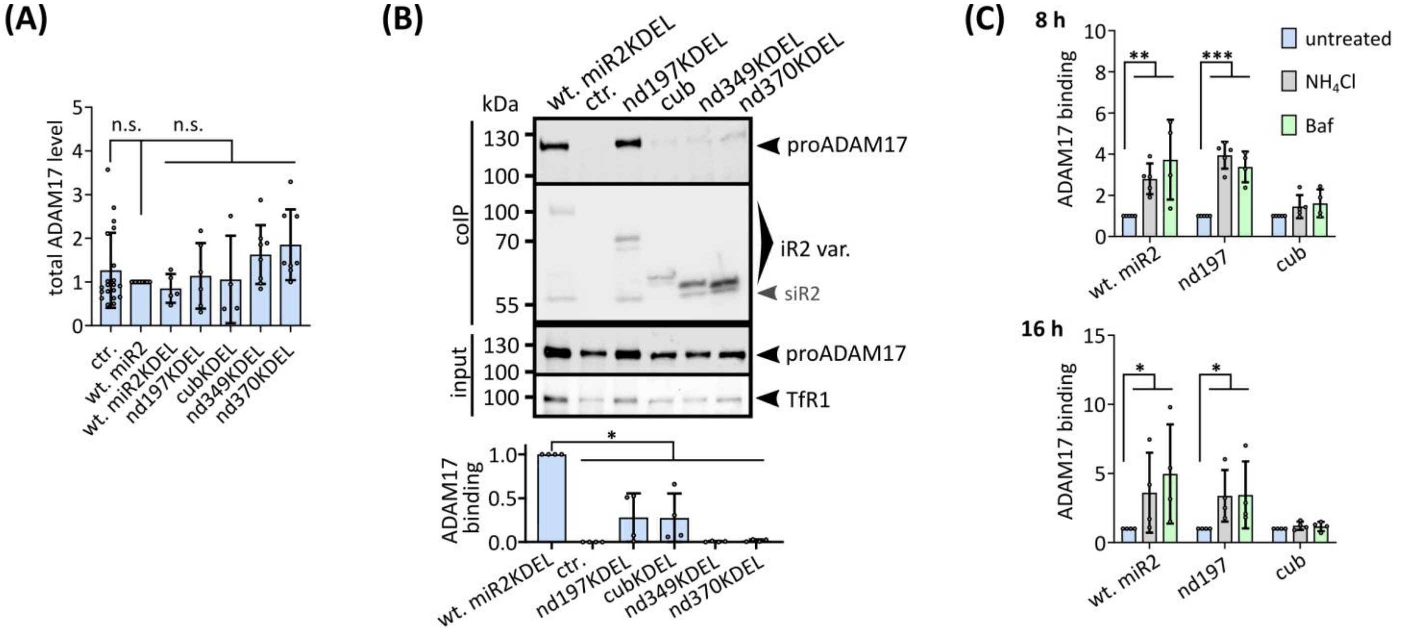

(A)
total ADAM17 level
n.s.
n.s.
ctr.
wt. miR2
wt. miR2KDEL
nd197KDEL
cubKDEL
nd349KDEL
nd370KDEL
(B)
kDa
wt. miR2KDEL
ctr.
nd197KDEL
cub
nd349KDEL
nd370KDEL
coIP
input
proADAM17
iR2 var.
siR2
proADAM17
TfR1
ADAM17 binding
*
(C)
8 h
16 h
ADAM17 binding
untreated
NH4Cl
Baf
**
***
*
*
wt. miR2
nd197
cub

Figure S4: (A) ADAM17 levels of immunoblot (Fig. 6C) were assessed by densitometric measurements and calculation of the ratio between total ADAM17 (ADAM17 plus proADAM17) divided by the respective input control. Values were normalised to the respective wt-iRhom2 sample. n > 5. **(B-C)** To analyse interaction between ADAM17 and the indicated iRhom2 variants, coIPs were performed using the iRhom variants (with HA tag) as bait. Immunoblotting and subsequent densitometric measurements were used to quantitatively analyse iRhom2-ADAM17 binding (ratio of co-precipitated ADAM17 and precipitated iRhom2) and normalised to wt-iRhom2. **(B)** MEFs from mice deficient for iRhom1 and iRhom2 stably expressing indicated iRhom2 variants with ER retention signal KDEL or GFP (ctr.) were used (n = 4). **(C)** iRhom-ADAM17 binding after lysosomal inhibition was analysed. Densitometric measurements of coIP immunoblot (Fig. 6D) (n = 4).
